# Human CD5^+^ Innate Lymphoid Cells Are Functionally Immature and Their Development from CD34^+^ Progenitor Cells Is Regulated by Id2

**DOI:** 10.3389/fimmu.2017.01047

**Published:** 2017-08-31

**Authors:** Maho Nagasawa, Kristine Germar, Bianca Blom, Hergen Spits

**Affiliations:** ^1^Department of Experimental Immunology, Academic Medical Center, University of Amsterdam, Amsterdam, Netherlands; ^2^Amsterdam Infection and Immunity Institute, Amsterdam, Netherlands; ^3^Department of Clinical Immunology and Rheumatology Center, Academic Medical Center, University of Amsterdam, Amsterdam, Netherlands

**Keywords:** innate lymphoid cells, human, development, Id2, CD5, CD5^+^ ILC

## Abstract

Innate lymphoid cells (ILCs) have emerged as a key cell type involved in surveillance and maintenance of mucosal tissues. Mouse ILCs rely on the transcriptional regulator Inhibitor of DNA-binding protein 2 (Id2) for their development. Here, we show that Id2 also drives development of human ILC because forced expression of Id2 in human thymic progenitors blocked T cell commitment, upregulated CD161 and promyelocytic leukemia zinc finger (PLZF), and maintained CD127 expression, markers that are characteristic for human ILCs. Surprisingly CD5 was also expressed on these *in vitro* generated ILCs. This was not an *in vitro* artifact because CD5 was also found on *ex vivo* isolated ILCs from thymus and from umbilical cord blood. CD5 was also expressed on small proportions of ILC2 and ILC3. CD5^+^ ILCs were functionally immature, but could further differentiate into mature CD5^−^ cytokine-secreting ILCs. Our data show that Id2 governs human ILC development from thymic progenitor cells toward immature CD5^+^ ILCs.

## Introduction

Innate lymphoid cells (ILCs) belong to a novel lymphoid cell subfamily. ILCs can be found throughout the body, particularly enriched in mucosal tissues and secondary lymphoid structures where they have been implicated as important regulators of mucosal homeostasis, host–microbiota interactions, initiation of protective immunity, inflammation, and tissue repair (1–3). ILCs have been categorized into three groups; ILC1 and NK cells, ILC2, and ILC3, based on their expression of typical transcription factors, cell surface markers and their cytokine production profile (4).

Recent papers have provided insight into the mechanisms of ILC development and the intermediate stages in mice and also in humans although our understanding of human ILC development lags that of mice. It has been suggested that human ILC development can occur not only in bone marrow, but also in secondary lymphoid tissues and the intestinal lamina propria (5, 6). ILC have also been found in the thymus which might imply that ILC also develop in the thymus. This notion is supported by the observation in mouse and human that the thymus contains bipotential T/NK progenitors (7–9), and it is plausible that such precursors may also develop into ILCs. Previously, we documented that lineage commitment of the bipotential thymic T/NK progenitors is dependent on the balanced expression levels of E-proteins, which are members of the basic helix-loop-helix (bHLH) transcription factor family, and their antagonist Id proteins (10, 11). Id2 is a member of a family of Helix-loop-helix factors consisting of four members, Id1, Id2, Id3, and Id4, which share the capacity of sequestering bHLH transcription factors, including E2A and HEB, which are needed for T and B cell development, and E2-2, required for development of plasmacytoid dendritic cells (pDC) (12–15). As a consequence, Id proteins can inhibit T and B cell and pDC development (10, 11, 16, 17). High levels of Id2 results in NK cell development by suppressing E2A and HEB activity, whereas a high ratio of E2A and HEB to Id2 favors T cell development (15). Id proteins inhibit T and B cell development at an early stage by blocking upregulation of RAG1 and RAG2 genes thereby prohibiting T cell receptor and B cell receptor rearrangements (10, 11, 16). This may set the stage for development of the precursors to ILCs. Indeed ILCs require expression of Id2 for their development, as its deficiency resulted in the complete absence of all ILCs in mice (18).

In addition to Id2, several other transcription factors have been identified that are required for mouse ILC development. Nuclear factor interleukin 3 (Nfil3) and thymocyte selection associated high mobility group box protein (TOX) are required for differentiation of the common lymphoid progenitor (CLP) into the integrin α4β7-expressing ILC progenitor α lymphoid precursor (αLP) (19–23). αLPs further differentiate into common helper ILC progenitors, which are progenitors of ILC1, ILC2, ILC3, and LTi cells, and promyelocytic leukemia zinc finger (PLZF) expressing ILC precursors (ILCp), which have lost the ability to develop into LTi cells, but retain potential to develop into the remaining ILC subsets (24, 25). The intermediate stages between stem cells and mature ILC in humans are less well defined. Recent studies in humans identified a potential ILCp displaying a RORγt^+^ CD34^+^ phenotype in tonsils and intestinal lamina propria that differentiated into IL-22 producing ILC3 (5). Another group identified a population of progenitor cells expressing RORγt and IL-1R1, the receptor for IL-1β, in secondary lymphoid tissues that differentiated into all ILCs, including NK cells, but not into T cells or DCs (6). More recently, a c-kit + ILCp was identified in peripheral blood of humans that *in vitro* could develop into all mature ILC subsets (26). As these cells were also found in various organs it was proposed that these circulating c-kit + ILC are able to home in the tissues and to develop into mature ILC in those tissues.

In the present study, we examined the capacity of Id2 to promote development of human ILC. We demonstrate that ectopic expression of Id2 blocked T cell differentiation, resulting in ILCs that expressed CD5 and intracellular (ic) CD3. *In vitro* generated ILCs expressing CD5 and icCD3 phenocopied ILCs that can be found *in vivo* in thymus and cord blood. *Ex vivo* isolated CD5^+^ non-T cells showed typical features of ILCs and displayed a functionally immature phenotype based on their inability to produce cytokines upon activation. CD5^+^ immature ILCs could be induced to differentiate into cytokine-producing CD5^−^ ILCs *in vitro*.

## Materials and Methods

### Monoclonal Antibodies and Cytokines

The following antibodies to human proteins were used. From BioLegend: fluorescein isothiocyanate (FITC)-conjugated anti-CD1a (HI149), anti-CD3 (OKT3), anti-CD4 (RPA-T4) anti-CD14 (HCD14), anti-CD16 (3G8), anti-CD19 (HIB19), anti-CD34 (581), anti-CD94 (DX22), anti-CD123 (6H6), anti-FcER1α (AER-37); phycoerythrin (PE)-conjugated anti-CD161 (HP-3G10), anti-NKp44 (P44-8), anti-IL-5 (JES1-39D10); Alexa Fluor 647-conjugated anti-NKp44 (P44-8); Alexa Fluor (AF) 700-conjugated anti-CD3 (UCHT1), anti-IL-17A (BL168); Allophycocyanin (APC)-conjugated anti-CD3 (OKT3), anti-CD56 (HCD56), anti-CD94 (DX22), anti-IL-13 (JES10-5A2); APC Cy7-conjugated anti-CD4 (OKT4); APC/Fire 750-conjugated anti-CD161 (HP-3G10); brilliant violet (BV) 421-conjugated anti-CD161 (HP-3G10), anti-CD3 (OKT3), anti-CD5 (UCHT2); BV510-conjugated anti-IFN-γ (4S.B3); BV650 streptavidin. From Becton Dickinson: FITC-conjugated anti-CD34 (581), anti-TCRαβ (IP26), ant-TCRγδ (B1), anti-CD8 (SK1); PE-CF594-conjugated anti-CD3 (UCHT1), anti-CRTH2 (BM16); AF647-conjugated anti-CRTH2 (CD294; BM16). From Beckman Coulter: PE-Cy7-conjugated anti-CD127 (R34.34); PE Cy5.5-conjugated anti-CD117 (104D2D1); PE-conjugated anti-CD1a (SFCI19Thy1A8). From Miltenyi: FITC-conjugated anti-BDCA2 (CD303; AC144); APC-Vio770-conjugated anti-CD5 (UCHT2). From eBiosciences: PE Cy7-conjugated anti-IL-22 (22URT1). From invitorogen: PE Cy5.5-conjugated anti-CD5(CD5-5D7). From NIH AIDS research program: purified anti-α4β7. Human cytokines: IL-2 was obtained from Novartis, IL-15, stem cell factor (SCF), IL-1β, IL-6, and TNF-α were obtained from R&D systems (Abingdon, UK). IL-7 and Flt3L were obtained from Pepro Tech, Inc. (Rocky Hill, NJ, USA).

### Cell Lines, Constructs, and Retrovirus Production

The naïve OP9 murine stromal cell line was kindly provided by Dr. T. Nakano (Osaka University, Osaka, Japan) OP9-Jag1, OP9-Jag2, and OP9–DL1 were generated as previously described (27). Id2 was isolated from the retroviral construct LZRS Id2 IRES GFP previously described (17) and subcloned into LZRS IRES mCherry by using restriction enzyme NotI (Roche, Germany). The empty construct was used in control transductions. Retroviral supernatant was obtained from transfected Phoenix-GALV packaging cells (28).

### Isolation of CD34^+^ Cells and ILCs from Postnatal Thymus (PNT) and Umbilical Cord Blood (UCB)

The use of PNT tissue and umbilical cord blood (UCB) was approved by the Medical Ethical Committee of the Academic Medical Center. Thymocytes were obtained from surgical specimens removed from children up to 3 years of age undergoing open heart surgery and UCB was collected with informed consent of the patients in accordance with the Declaration of Helsinki. The tissue was disrupted by mechanical means and pressed through a stainless steel mesh to obtain a single-cell suspension, which was left overnight at 4°C. The next day, thymocytes were isolated from a Ficoll–Hypaque density gradient (Lymphoprep; Axis-Shield). Subsequently, CD34^+^ cells were enriched by immunomagnetic cell sorting, using a CD34 cell separation kit (Miltenyi Biotec, Bergisch Gladbach, Germany). The CD34^+^ thymocytes were stained with Abs against CD34, CD1a, CD56, and BDCA2. CD34^+^CD1a^−^CD56^−^BDCA2^−^ cells, further referred to as CD34^+^CD1a^−^, were sorted to purity on a FACSAria (BD Biosciences). For the isolation of ILCs from PNT and UCB, mononuclear cells isolated from a Ficoll–Hypaque density gradient (Lymphoprep; Axis-Shield) were positively selected by labeling with PE-conjugated anti-CD161 (described above) plus anti-PE microbeads (Miltenyi). The CD161^+^ cells were stained with Abs against Lineage (CD1a, CD3, CD4, CD8, CD14, CD19, CD16, CD34, CD94, CD123, TCRαβ, TCRγδ, FcER1α), CD161, CD127, CD117, CRTH2, NKp44, and CD5. Cells were sorted on a FACSAria, purity of the sorted cells in all experiments was >99%.

### Retroviral Transduction and Differentiation Assay

For transduction experiments, CD34^+^CD1a^−^ postnatal thymocytes were cultured overnight in Yssel’s medium (29) with 5% normal human serum, 20 ng/ml SCF and 10 ng/ml IL-7. The following day cells were incubated for 6–7 h with virus supernatant in retronectin-coated plates (30 µg/ml; Takara Biomedicals, Shiga, Japan). The development of ILCs and NK cells was assessed by coculturing the mixture of transduced and non-transduced CD34^+^CD1a^−^ progenitor cells with OP9 cells in MEMα medium (Life Technologies, Carlsbad, CA, USA) with 20% FCS (Hyclone Laboratories, Logan, UT, USA), 5 ng/ml SCF, 5 ng/ml IL-7, and 5 ng/ml FLT3L. 0.5 ng/ml IL-15 was added at the onset of the culture, the medium containing IL-15 was refreshed every week. Flow cytometric analyses were performed on a LSRII FACS analyzer (BD Biosciences); electronic gating was performed using FlowJo (Tree Star, Ashland, OR, USA). Numbers in each dot plot represent the percentage of cells in each quadrant. The fold expansion in absolute cell numbers was calculated using Microsoft Excel 2007 (Microsoft, Redmond, WA, USA) on the basis of total numbers of cells harvested from the cultures, percentages of transduced cells, and percentages of each population corrected for the number of input cells.

### Quantitative Real-time PCR

Total RNA was extracted with a NucleoSpin RNA XS kit (Macherey-Nagel) according to the manufacturer’s instructions. cDNA was synthesized with a High-Capacity cDNA Archive kit (Applied Biosystems). PCRs were performed in a Bio-Rad iCycler (Bio-Rad, France) with IQ SYBR Green Supermix (Bio-Rad, France) using the following primer sets or otherwise as previously described (30) Id2 forward 5′-TTGTCAGCCTGCATCACCAGAG-3′; Id2 reverse 5′-AGCCACACAGTGCTTTGCTGTC-3′; PLZF forward 5′-GAGCTTCCTGATAACGAGGCTG-3′; PLZF reverse 5′-AGCCGCAAACTATCCAGGAACC-3′; Nfil3 forward 5′-TGGAGAAGACGAGCAACAGGTC-3′; Nfil3 reverse 5′-CTTGTGTGGCAAGGCAGAGGAA-3′; TOX forward 5′-CGCTACCTTTGGCGAAGTCTCT-3′; TOX reverse 5′-CTGGCTCTGTATGCTGCGAGTT-3′. Bio-Rad CFX Manager 3.1 software was used for quantification of expression. All samples were normalized to the expression of GAPDH and results are presented in arbitrary units.

### ILC Differentiation, Single Cell Cloning, and Expansion

For differentiation assays, isolated ILCs were cultured in Yssel’s medium supplemented with 1% (v/v) human AB serum (Invitrogen) for 5–7 days. Cytokines used in these assays were IL-2 (10 U/ml), IL-7 (10 ng/ml), IL-1β (50 ng/ml), IL-6 (50 ng/ml), and TNF-α (50 ng/ml). Single cell or bulk ILCs were expanded *in vitro* by culturing with 2 × 10^6^/ml irradiated (25 Gy) allogenic peripheral blood mononuclear cells, 2 × 10^5^/ml irradiated (50 Gy) JY Epstein–Barr virus-transformed B cells, phytohemagglutinin (1 µg/ml; Oxoid), IL-2 (100 U/ml), and IL-7 (10 ng/ml) in Yssel’s medium.

## Results

### ILCs Are Present in Thymus and Express Id2

**Figure 1:**
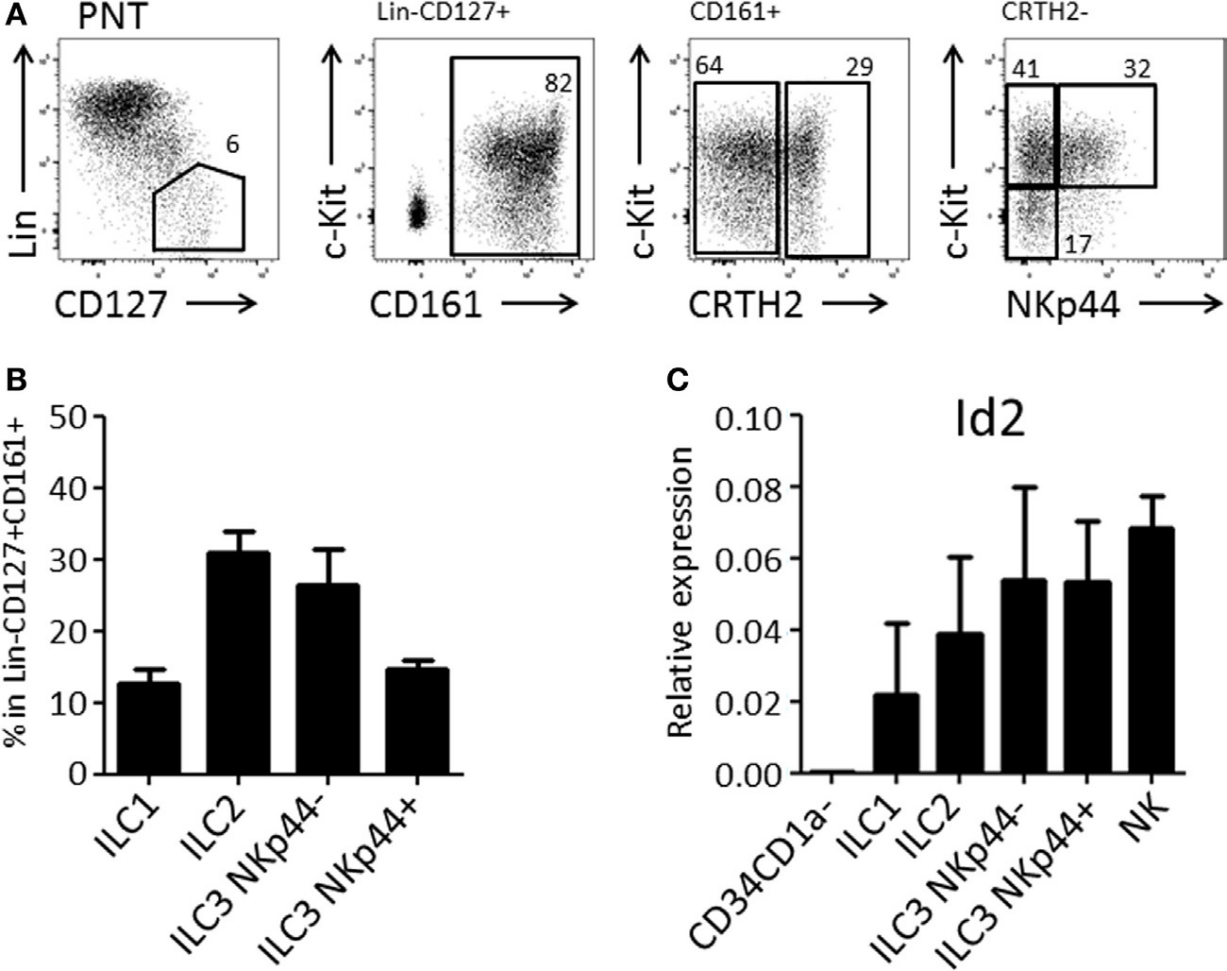
Human postnatal thymus (PNT) contains all Innate lymphoid cell (ILC) subsets. **(A)** Gating strategy by flow cytometry of thymic ILC subsets. CD161 MACS-enriched thymocytes were stained with Lineage (CD1a, CD3, CD4, CD8, CD14, CD16, CD19, CD34, CD94, BDCA2, TCRαβ, TCRγδ, FcεRI), CD127, CD161, CRTH2, c-Kit, and NKp44. **(B)** Frequency (%) of ILC subsets in Lin^−^CD127^+^CD161^+^ ILC population. The data shown are average of five donors. **(C)** qPCR analysis of Id2 mRNA expression in thymic ILC subsets. The data shown are average of two donors. CD34^+^CD1a^−^ progenitor cells and NK cells were isolated from PNT and used for negative and positive controls, respectively.

We and others have demonstrated that the thymus contains bispecific T/NK cell progenitors (7–9, 15). In humans, these cells are contained within CD34^+^CD1a^−^CD5^+^ cells (9). We expected that thymic T/NK cell progenitors would also be able to develop into ILC within the thymus. Therefore, we first investigated the presence of ILC subsets in the human thymus. We observed that human thymus contained ILCs at a frequency of approximately 1 in 100,000 total thymocytes. All ILC subsets, ILC1, ILC2, and ILC3 (both NKp44^+^ and NKp44^−^) were present (Figures 1A,B) and that all subsets expressed higher levels of Id2 as compared to CD34^+^CD1a^−^ thymic progenitor cells (Figure 1C).

### Id2 Induces Differentiation of CD161-Expressing ILCs

**Figure 2:**
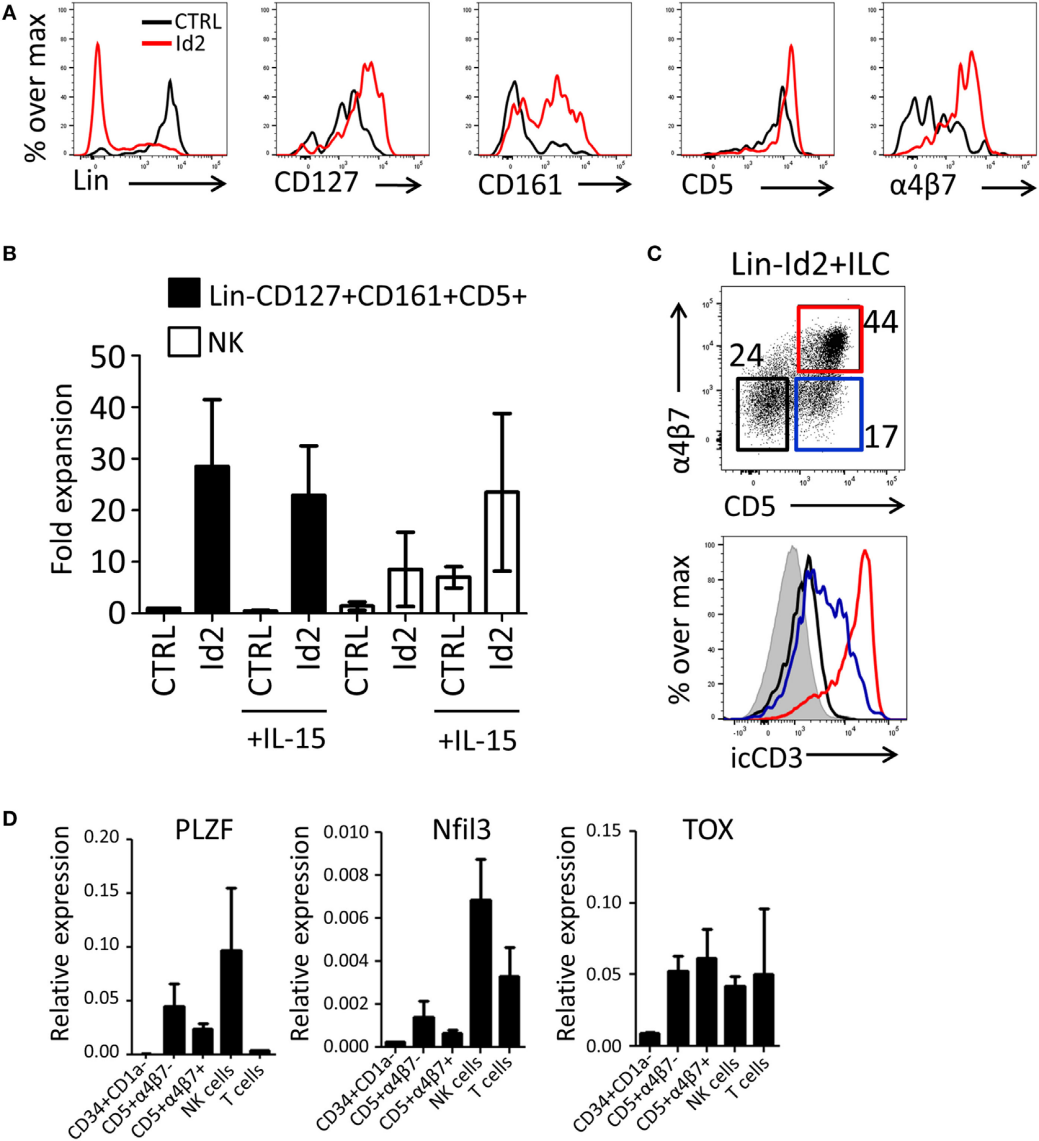
Id2 induces development of Lin^−^CD127^+^CD161^+^CD5^+^ cells from PNT CD34^+^CD1a^−^ cells. **(A)** Cell surface molecule expression on control or Id2-transduced cells after 7 days cultured on OP9 cells. **(B)** Fold expansion of control or Id2-transduced Lin^−^CD127^+^CD161^+^CD5^+^ and NK cells with or without IL-15. The cell number of control transduced Lin^−^CD127^+^CD161^+^CD5^+^ cells in the culture without IL-15 was set as 1. NK cells were determined by their cell surface expression of CD56 and low CD127. The data shown are an average of three independent experiments at day 13 of OP9 coculture. **(C)** Intracellular CD3 (clone OKT3) staining of Id2^+^Lin^−^CD127^+^CD161^+^ cells generated on OP9-Jag1 after 7 days. The population is further divided based on the expression of surface CD5 and α4β7. icCD3 histogram indicated in red: CD5^+^α4β7^+^, blue: CD5^+^α4β7^−^, black: CD5^−^α4β7^−^, gray filled: isotype control. **(D)** qPCR analysis of promyelocytic leukemia zinc finger (PLZF), Nfil3, and TOX mRNA expression in Id2^+^Lin^−^CD127^+^CD161^+^CD5^+^ cells generated on OP9–Jag1 and sorted at day 7 of coculturing. PNT CD34^+^CD1a^−^ cells and NK cells were used as negative and positive controls, respectively. All qPCR values presented are relative to GAPDH expression.

Previously, we have demonstrated that thymic progenitors that overexpress Id2 develop into NK cells in response to IL-15 (15). Here, we investigated the effect of forced expression of Id2 in thymic precursors on ILC development. Id2-transduced CD34^+^CD1a^−^ cells were cocultured with OP9 mouse stromal cells in the presence of IL-7, SCF, and FLT3L. We observed that Id2 increased the development of ILC-like cells that expressed CD127, CD161, and integrin α4β7 (Figures 2A,B), both in proportion as well as in absolute cell numbers. Consistent with our previous report (15), Id2 inhibited differentiation into T cells and pDC, showing that the balance between E-proteins and Id2 is important for the cell lineage decision between T cells, pDC, and NK/ILC cells (14, 15, 17). Like CD127, CD5 was expressed on virtually all thymic progenitor cells and no difference in expression of these antigens was observed between control and Id2 conditions (Figure 2A). Thus, Id2 transduction resulted in the expansion of a lineage (CD1a, CD3, CD4, CD8, CD94, BDCA2) negative CD5^+^CD161^+^CD127^+^ cell population resembling ILCs (Figure 2A; Figure S1A in Supplementary Material). While all control transduced ILCs expressed c-Kit, only a fraction of the *in vitro* generated Id2^+^ ILCs expressed c-Kit, whereas no CRTH2^+^ ILC2 or NKp44^+^ ILC3 were detected (Figure S1A in Supplementary Material). This CD5^+^ ILC-like cell population was also generated on OP9 cells expressing the Notch ligands Jagged1 (Jag1), Jagged2 (Jag2) or delta-like 1(DL1), with the highest expansion rate when cells were cultured on OP9-Jag1 (Figures S1A,B in Supplementary Material), indicating that the Notch ligand Jag2 can affect *in vitro* expansion of Id2^+^ ILCs, but Notch ligands have little effect on their phenotype. Id2^+^(mCherry^+^)Lin^−^CD127^+^CD161^+^ cells can be divided into three populations based on their CD5 and α4β7 expression (Figure 2C). The Id2^+^Lin^−^CD127^+^CD161^+^ cells expressing both CD5 and α4β7 lacked cell surface (s) expression of CD3, but expressed high intracellular (ic) CD3 staining, while the cells with CD5, but no α4β7 showed lower icCD3 staining. Our data indicate that whereas Id2 inhibits TCR rearrangement and expression, induction of intracellular expression of CD3 is not prevented by Id2. As ectopic expression of Id2 completely blocks development of T cells by preventing TCR rearrangements (10, 11), sCD3^−^icCD3^+^CD5^+^ should be non-T cells. CD4, which is expressed on immature single positive T cell progenitors (31), was not detected on Id2^+^CD5^+^ ILC (Figure S1D in Supplementary Material). Id2-induced ILCs expressed higher levels of PLZF, Nfil3, and TOX transcripts when compared with CD34^+^CD1a^−^ progenitor cells, especially the CD5^+^α4β7^−^ ILC population, which had slightly higher expression of PLZF and Nfil3 compared to the CD5^+^α4β7^+^ population (Figure 2D). Nfil3 and TOX expression were not restricted to ILCs as high expression was also detected in both NK and T cells, but PLZF expression was hardly detected in T cells, consistent with the notion that PLZF expressing Id2^+^Lin^−^CD127^+^CD161^+^CD5^+^ cells are indeed ILCs.

The expansion of NK cells by Id2 is strongly increased in the presence of IL-15 (Figure 2B). In contrast, no effect of IL-15 was observed on the expansion of the Lin^−^CD1a^−^CD5^+^CD161^+^CD127^+^ ILC-like cell population (Figure 2B) indicating that differentiation and expansion of Lin^−^CD1a^−^CD5^+^CD161^+^CD127^+^ cells are independent of IL-15. To substantiate this finding, we investigated whether the *in vitro* generated CD5^+^ ILCs have the capacity to develop further into NK cells. We purified the Id2^+^Lin^−^CD5^+^CD127^+^CD161^+^ cells generated on OP9-Jag1 and cultured these cells with OP9 cells with or without IL-15. We observed no upregulation of the NK cell markers CD94 or CD56. Furthermore, CD127 was not downregulated by IL-15 (Figure S1C in Supplementary Material). These results indicate that Id2^+^Lin-CD5^+^CD161^+^CD127^+^ cells are not precursors of NK cells.

### CD5^+^ ILCs Are Present in the PNT and UCB

**Figure 3:**
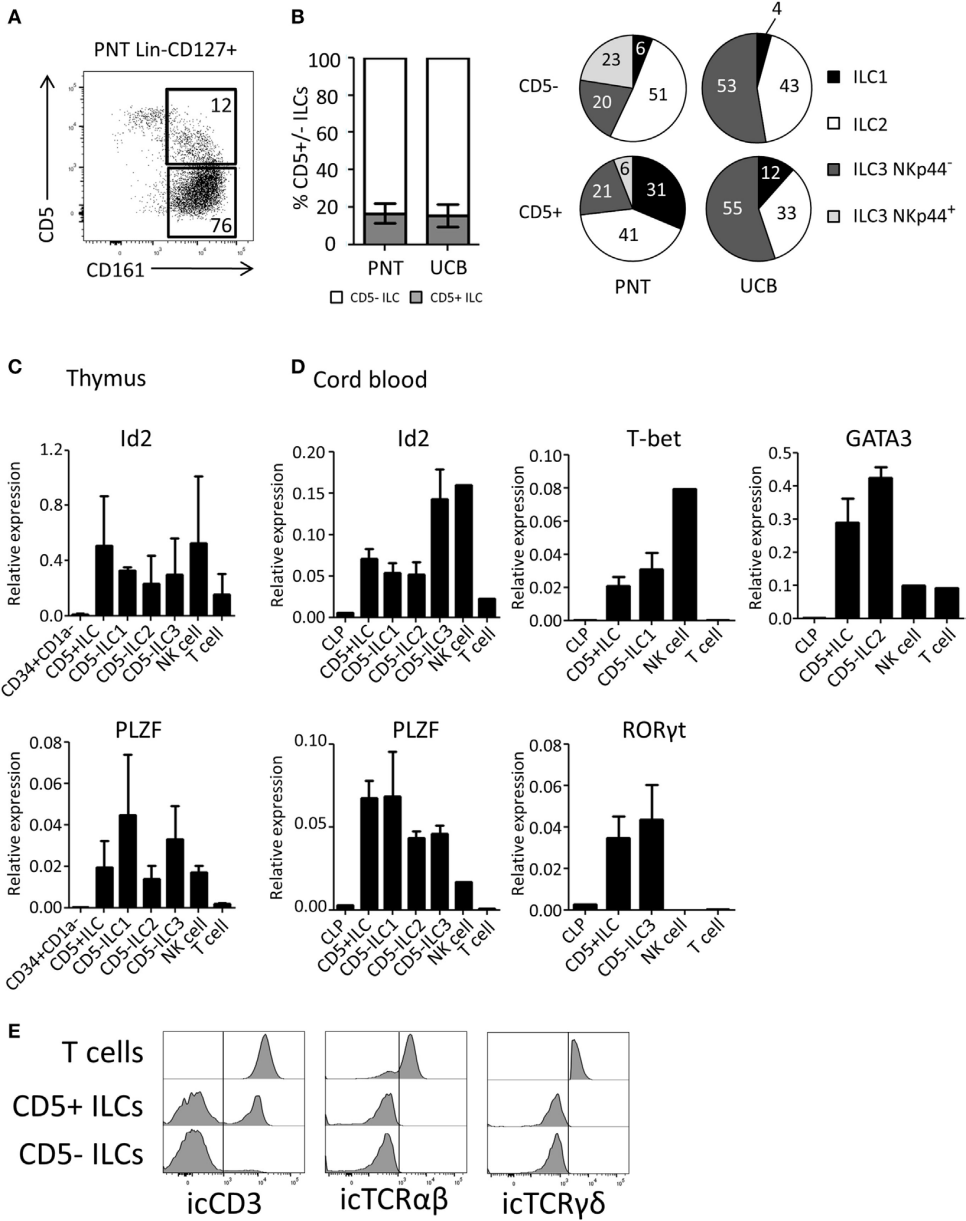
CD5^+^ ILCs are present in the postnatal thymus (PNT) and umbilical cord blood. **(A)** Flow cytometry of PNT Lin^−^CD127^+^ showing CD5^+^ ILCs **(B)** Percentage of CD5^+^ cells within total innate lymphoid cell (ILC) population (Lin^−^CD127^+^CD161^+^) in PNT and umbilical cord blood (UCB). Pie chart indicates the proportion of ILC subsets in the CD5^+^ or CD5^−^ fraction of PNT and UCB. Summary of PNT *n* = 4 and UCB *n* = 5 **(C)** Id2 and PLZF mRNA expression levels CD5^+^ ILCs and CD5^−^ ILC subsets isolated from PNT were determined by qPCR. CD34^+^CD1a^−^ progenitors, NK cells and T cells were isolated from the thymus and used as a reference. The data shown are average of three donors. **(D)** Id2, PLZF, T-bet, Gata3, and RORγt mRNA expression levels in CD5^+^ ILCs and CD5^−^ ILC subsets isolated from UCB were determined by qPCR common lymphoid progenitor (CLP), NK cells, and T cells isolated from UCB were used as a reference. The data shown are average of three donors. All qPCR values presented are relative to GAPDH expression. **(E)** UCB CD5^+^ and CD5^−^ ILCs were intracellularly stained with anti-CD3 (clone OKT3), anti-TCRαβ, and anti-TCRγδ antibodies. T cells isolated from UCB were used as positive control. icTCRγδ histogram shown is gated on icTCRγδ positive fraction of control T cells.

We next investigated the expression of CD5 on *ex vivo* isolated ILC subsets. We observed that on average 17% of the total thymic ILC population (Lin^−^CD127^+^CD161^+^) expressed CD5 (Figure 3A). Similar percentages of CD5^+^ ILCs were detected in UCB (Figure 3B). The CD5 expression level on ILCs was lower compared to T cells (Figure S2B in Supplementary Material). In contrast to *in vitro* generated Id2^+^CD5^+^ ILCs, a small proportion of *ex vivo* CD5^+^ ILCs expressed CRTH2 and NKp44 in addition to c-Kit, which indicates that CD5 can be expressed on all ILC subsets (Figure 3B). In addition, we observed a minor population of α4β7^+^ cells among CD5^+^ ILCs (Figure S2A in Supplementary Material). We tested CD5^+^ ILCs from thymus and UCB for ILC related gene expression by qPCR. Similar to CD5^−^ ILCs, CD5^+^ ILCs expressed Id2 and PLZF (Figures 3C,D). In addition, representative transcription factors for each ILC subset, namely T-bet (ILC1), GATA3 (ILC2) and RORγt (ILC3), were expressed at similar levels in the UCB CD5^−^ and CD5^+^ ILCs (Figure 3D). In contrast to CD5^+^ ILCs, CD5^+^CD1a^+^ thymic T cell progenitors lacked Id2 and PLZF (Figure S2C in Supplementary Material) consistent with the notion that CD5^+^ ILCs are not T cell precursors. We next considered the possibility that CD5^+^ ILCs are in fact mature T cells, which have downregulated their T cell receptor as CD5 is commonly known to be expressed on mature T cells (32). We found, however, that although part, but not all, of CD5^+^ ILCs from UCB expressed icCD3, no icTCRαβ or icTCRγδ expression was detected (Figure 3E), enforcing the notion that CD5^+^CD127^+^CD161^+^ cells that also express Id2 and PLZF (which was not expressed in UCB T cells) are ILCs and not T cells.

### Thymic and Cord Blood CD5^+^ ILCs Are Functionally Immature

**Figure 4:**
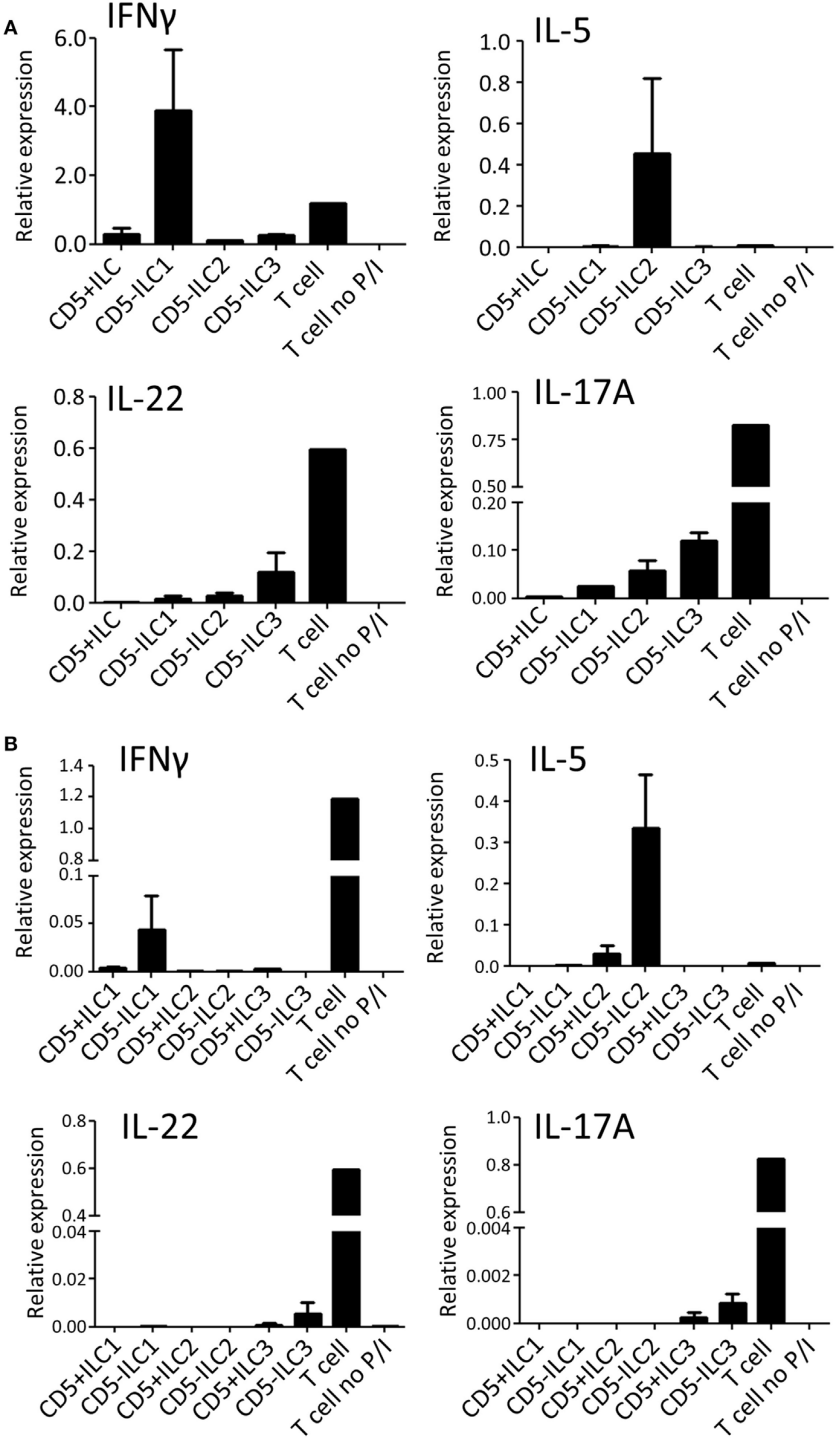
CD5^+^ ILCs display a functionally immature phenotype. **(A)** qPCR analysis of cytokine mRNA expression levels of total PNT CD5^+^ ILC compared to CD5^−^ ILCs after 6 h PMA/ionomycin (P/I) stimulation. Tonsil T cells were used as stimulated and unstimulated references. The data shown are average of two independent experiments. **(B)** qPCR analysis of cytokine mRNA expression levels of UCB CD5^+^ ILC subsets compared to CD5^−^ ILC subsets after 6 h P/I stimulation. Tonsil T cells were used as stimulated and unstimulated references. The data shown are average of two independent experiments. All qPCR values presented are relative to GAPDH expression.

To assess their ability to produce cytokines, CD5^+^ ILCs from thymus were sorted and stimulated with PMA/Ionomycin and analyzed for cytokine gene expression by qPCR (Figure 4A). CD5^−^ ILC subsets were also isolated from the thymus and used as a reference. Notably, CD5^+^ ILCs did not express any of the ILC signature cytokine genes after stimulation, whereas CD5^−^ ILC1 expressed IFNγ, CD5^−^ ILC2 expressed IL-5 and CD5^−^ ILC3 expressed IL-17A and IL-22. All ILCs, including CD5^+^ ILCs, expressed IL-2, but at lower levels compared to T cells isolated from tonsils (Figure S3A in Supplementary Material). We further investigated the expression of cytokine-encoded genes by UCB CD5^+^ ILCs. Similarly to thymic CD5^+^ ILCs, UCB CD5^+^ ILC either did not respond to stimulation or expressed lower levels of cytokine genes than CD5^−^ ILC (Figure 4B). ILC3 related cytokine gene expression was low in both CD5^+^ and CD5^−^ ILC, which is in line with our observation that adult peripheral blood ILC3s lack the ability to produce significant amounts of IL-17A and IL-22 upon activation with PMA and ionomycin (Figure S3B in Supplementary Material). IL-2 expression was also low in all UCB ILCs compared to T cells from tonsil (Figure S3A in Supplementary Material). Hence, CD5^+^ ILCs appear to be functionally immature as compared to CD5^−^ ILCs.

### UCB CD5^+^ ILCs Differentiate into CD5^−^ ILCs

**Figure 5:**
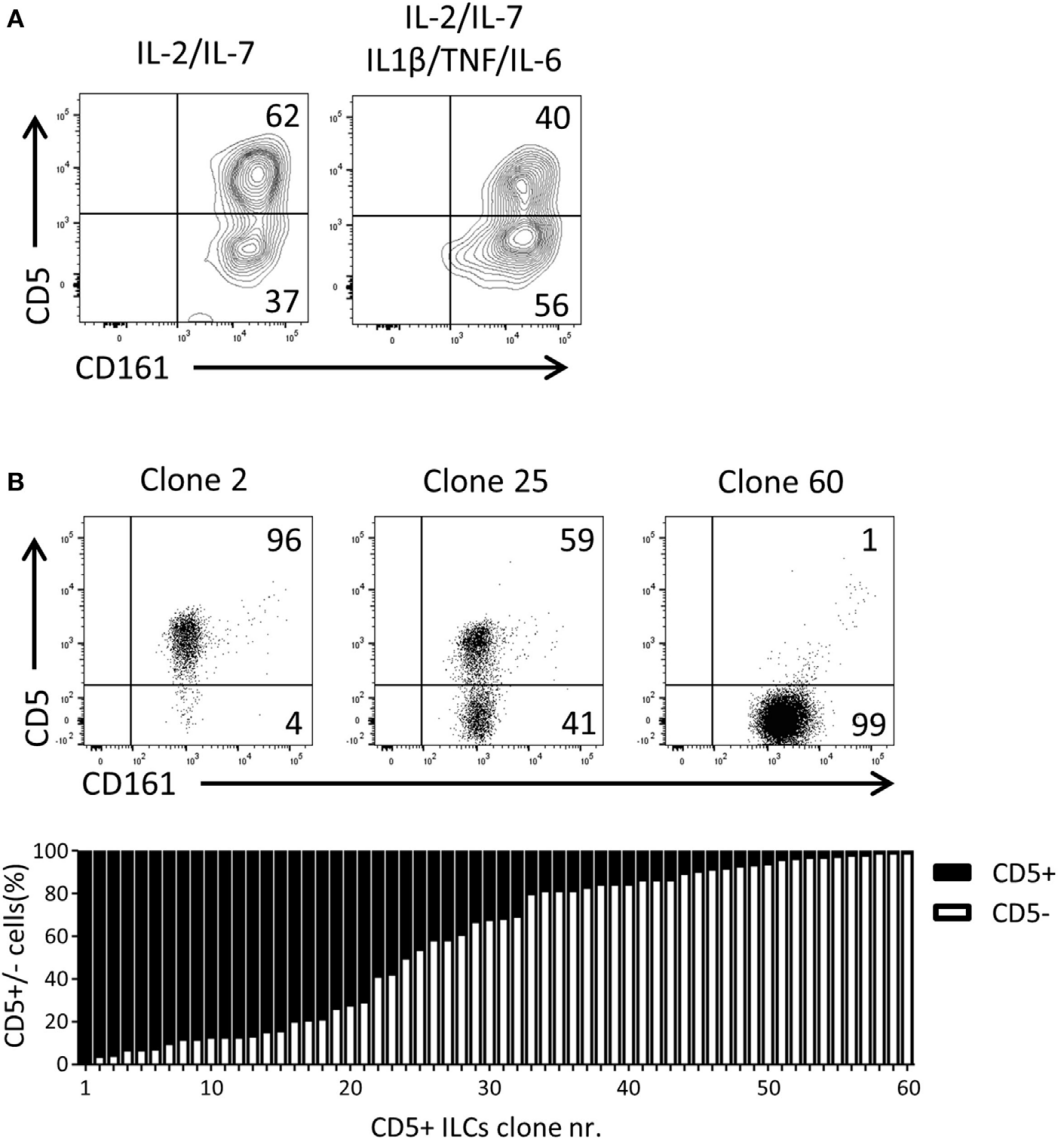
UCB CD5^+^ ILCs differentiate into CD5^−^ ILCs. **(A)** Flow cytometry analysis of CD5^+^ ILC phenotype after 7 days in culture with indicated cytokines. The plots showed here is pre-gated for CD3^−^CD94^−^ cells. CD5^+^ ILCs were isolated from UCB and cultured with IL-2 (10 U/ml) and IL-7(10 ng/ml) or IL-2, IL-7, IL-1β (50 ng/ml), IL-6 (50 ng/ml), and TNFα (50 ng/ml). The data shown are a representative of four independent experiments. **(B)** Flow cytometry analysis of three representative CD5^+^ ILC clones (top), frequency of CD5 expression (bottom).

Given that CD5^+^ ILCs presented as functionally immature ILCs upon stimulation, we asked whether CD5^+^ ILCs may be precursors of CD5^−^ ILCs. To test the differentiation capacity of CD5^+^ ILCs, total CD5^+^ ILCs were highly purified from cord blood and cultured for 7 days in the presence of IL-2 and IL-7. We observed that part of CD5^+^ ILCs lost CD5 expression (Figure 5A). This differentiation was also induced when the cells were cultured with IL-1β, TNFα, and IL-6, cytokines typically elevated in the inflammatory state. Even though the cells were isolated at high purity, we still cannot exclude the possibility that CD5^−^ ILCs appearing in those culture conditions are derived from a minor contamination of CD5^−^ ILCs. Thus, we deposited single CD5^+^ ILCs in wells of a microtiter culture plate and generated clones in the presence of irradiated allogenic peripheral blood mononuclear cells and JY cells (feeder cells), IL-2 and IL-7. After 3 weeks of culturing, 65 out of 360 wells showed cell growth equivalent to 17% plating efficiency, making it highly likely that the starting cell number was indeed 1 cell per well as previously demonstrated (33). Among those 65 clones, 5 clones exhibited sCD3 expression (data not shown) and were excluded from further analysis. There were three types of CD5^+^ ILC-derived clones observed; those which remained CD5^+^, those which gave rise to both CD5^+^ and CD5^−^ ILCs, and those which became CD5^−^ ILCs (Figure 5B). The frequency of those three phenotypes was equally distributed among the clones. This result strongly indicates that CD5^+^ ILCs can indeed differentiate into CD5^−^ ILCs, and confirmed that CD5^−^ ILCs appearing in differentiation and proliferation assays were derived from CD5^+^ ILCs.

### UCB CD5^−^ ILC2s Are Functionally Competent and Derive from CD5^+^ ILC2s

**Figure 6:**
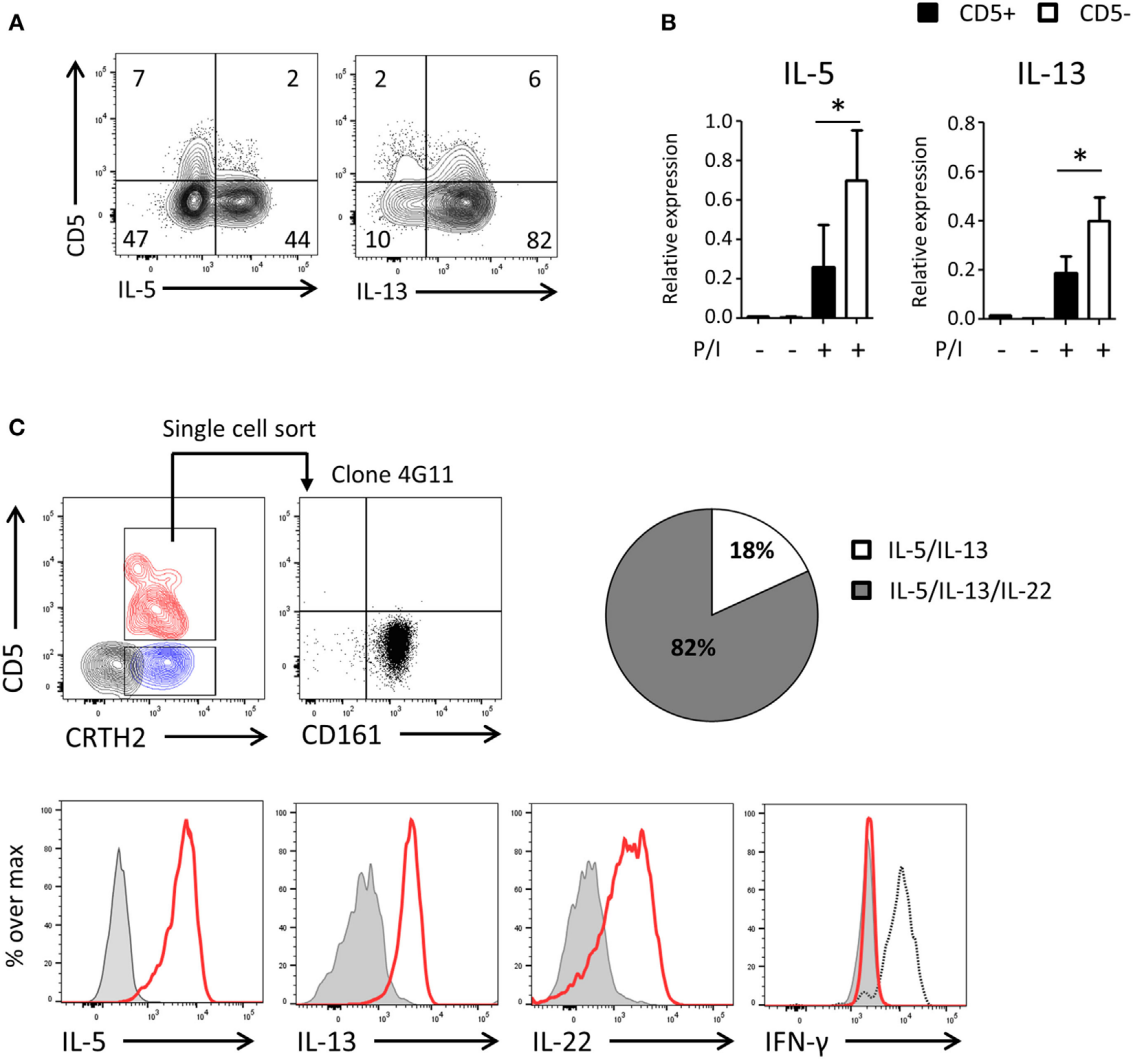
UCB CD5^−^ ILC2s are functionally competent and derive from CD5^+^ ILC2s. **(A)** Cytokine production of bulk CD5^+^ ILC2 after 2 weeks of coculturing with the irradiated allogenic peripheral blood mononuclear cells and JY cell (feeder cells) with IL-2 (100 U/ml) and IL-7 (10 ng/ml). Total cells were stimulated with P/I for 6 h. **(B)** qPCR analysis of cytokine expression level of CD5^+^ ILC2 derived CD5^+^ ILC2s and CD5- ILC2s. Cells were sorted from the feeder cells coculture with IL-2 (100 U/ml) and IL-7 (10 ng/ml) after 2 weeks and unstimulated or stimulated with P/I for 6 h (*n* = 5). *P < 0.05 (Student’s *t*-test) **(C)** Single CD5^+^ CRTH2^+^ ILC2 cell was sorted and cultured in feeder cells with IL-2 (100 U/ml) and IL-7 (10 ng/ml) for 2 weeks and cytokine production was evaluated after stimulated with P/I for 6 h. Counter plot (left top): CD5^+^ CRTH2^+^ ILC2 (red), CD5^−^CRTH2^+^ ILC2 (blue) and CD5^−^ CRTH2^−^ ILC1 (black). Dotplot (right top): CD5 and CD161 expression on one representative clone (4G11). Histogram: clone (red), isotype control (gray filled), and IFN-γ positive control (black dashed). Pie chart indicate the frequency of clones producing IL-5 and IL-13 or IL-5 and IL-13 and IL-22.

To assess the functionality of CD5^−^ ILCs derived from CD5^+^ precursors, we cultured highly purified cord blood CD5^+^ ILC2 with feeder cells in the presence of IL-2 and IL-7 (Figure 6A) and after 2 weeks determined the production of cytokines and expression of cytokine transcripts by CD5^+^ and CD5^−^ ILC2s following PMA/ionomycin stimulation. A higher frequency of cytokine-producing cells in the CD5^−^ fraction compare to the CD5^+^ fraction was observed and cytokine gene expression levels of CD5^+^ ILC2s and CD5^−^ ILC2s, derived from CD5^+^ ILC2s, were significantly different (Figures 6A,B). To further corroborate that CD5^+^ ILC2 can differentiate into mature CD5^−^ ILC2 we performed single cell cloning experiments starting from ILCs that expressed CRTH2^+^ (Figure 6C). Consistent with the previous experiments, most of the clones had lost CD5 and were able to produce type 2 cytokines upon stimulation with PMA and ionomycin. In addition to type 2 cytokines, we have also tested type 1 (IFN-γ) and type 3 (IL-17A and IL-22) cytokines. There were no IFN-γ nor IL-17A producing clones observed, suggesting that CD5^+^ LC2s are ILC2 committed precursors. We observed that many clones produced IL-22 consistent with earlier observations on cultured ILC2 from peripheral blood (34). These results together indicate that CD5^+^ ILC2s can differentiate into functionally competent CD5^−^ ILC2.

## Discussion

Our data show that expression of Id2 in human progenitor cells favors development of ILCs while inhibiting T cell and pDC development (10, 11, 14, 15, 17). One of the mechanisms by which Id2 inhibits T cell development is preventing TCR gene rearrangements (10). As CD5 was described to be a marker that distinguishes T cells from ILCs (35) it was unexpected to observe that under conditions in which TCR gene rearrangements and thus T cell development is blocked, CD5^+^ ILCs are generated. These CD5^+^ ILCs cannot be T cells that have downregulated the expression of TCR, because Id2 prevents TCR gene rearrangements (10). CD5 expression on ILCs that express ectopic Id2 is not an *in vitro* artifact, because CD5^+^ ILCs were also found when isolated *ex vivo* from thymus and UCB. These cells are distinct from CD5^+^ T-cell progenitors as they express Id2, which precludes T-cell development, and lack expression of CD1a (12). Moreover, CD5^+^ ILC expressed markers that are characteristic of ILCs, including CD127, CD161, Id2, and PLZF. As it was shown that Id2 promoted expression of PLZF during invariant NKT cell development in the mouse (36), our findings support the notion that Id2 may regulate the expression of PLZF to drive human ILC lineage development, but it should be noted that PLZF may be differentially regulated in mouse and human ILC, because unlike mouse ILCs (24) PLZF is also expressed in mature ILCs in humans (37). A proportion of CD5^+^ ILCs express icCD3, similar to the CD5^+^ ILC generated from Id2 overexpressing progenitor cells, but did not express TCRαβ or TCRγδ proteins in the cytoplasm consistent with the fact that Id2 inhibits TCR gene rearrangement. Together with our data of Id2 overexpression, we conclude that CD5^+^ ILCs are not T cells in disguise, but rather are *bona fide* ILCs. Further confirmation is provided by the observation that almost all clones derived from CD5^+^ ILCs did not express the TCR/CD3 complex on their cell surface. *In vitro* generated Id2^+^CD5^+^ ILCs did not further differentiate into NK cells in the presence of IL-15, a cytokine known to be essential for NK cell development (38, 39), suggesting that Id2^+^CD5^+^ ILCs are downstream of bipotent NK/ILCp. About half of the *in vitro* generated CD5^+^Id2^+^ ILC expressed α4β7 that is also expressed on mouse common ILCp (23), suggesting that CD5 is induced early in ILC development.

Close inspection of CD5 expression on thymic and UCB ILCs revealed that CD5 is also expressed on a minority of ILCs that have hallmarks of ILC2 and ILC3. *Ex vivo* CD5^+^ ILC1, ILC2, and ILC3 were unable to produce cytokines after stimulation with PMA and ionomycin, suggesting that they are functionally immature. Indeed, functional studies and cloning experiments clearly demonstrate that CD5^+^CRTH2^+^ ILC2, which produced very low amounts of type 2 cytokines can differentiate into CD5^−^ ILC2 capable of producing high amounts of cytokines. Together, our data indicate that CD5 is expressed on early ILC progenitor cells and on immature ILC1, ILC2, and ILC3. Functional maturation is accompanied by downregulation of CD5. A recent report presented evidence for the existence of c-Kit + multipotent ILCp in peripheral blood, which differentiates into all cytokine-producing ILC subsets (26). These cells were selected for absence of CD5. However, because CD5^+^ ILCs express one log less CD5 than T cells (Figure S2B in Supplementary Material), it might be possible that Lim et al. by adding anti-CD5 to their antibody cocktail to remove non-ILCs, depleted CD5^+^ T cells, but not CD5^+^ ILC.

CD5^+^ ILCs are present in the adult peripheral blood, spleen, lung, and bone marrow (data not shown) indicating that immature CD5^+^ ILCs are not restricted to infants. We observed that CD5^+^ ILCs were not present in inflamed nasal polyps (data not shown) of patients suffering chronic rhinosinusitis with nasal polyps in which ILC2 accumulate (30, 34), suggesting that immature CD5^+^ ILCs fully differentiate into mature CD5^−^ ILCs under inflammatory conditions. This notion is supported by our observation that, pro-inflammatory cytokines IL-1β, IL-6, and TNF-α promoted differentiation of CD5^+^ ILCs into CD5^−^ ILCs *in vitro*.

It has been reported that SCID patients with IL-2 receptor gamma or JAK3 deficiency not only lack T cells, B cells, and NK cells, but also ILC subsets. After hematopoietic stem cell transplantation in these patients, T cells, B cells, and ILC1—but no other ILC subsets including NK cells—were reconstituted (40). Interestingly, these reconstituted ILC1 expressed CD5. Given our suggestion that CD5 expression represents an immature stage of ILCs, this may indicate that the CD5^+^ ILC1s in these patients are arrested from further differentiation into mature ILCs. Recently CD4^+^ ILC1 were found in peripheral blood (41). We confirmed those findings and found that those CD4^+^ ILC1 also expressed CD5 [(42) and Nagasawa, unpublished observation]. However, in contrast, the cord blood and thymic CD5^+^ ILCs described here, which express CD161, lacked CD4. The relationship between the immature CD5^+^ ILCs described here and the CD4^+^ CD5^+^ ILC1 described previously has yet to be fully established.

## Ethics Statement

The use of postnatal thymus (PNT) tissue and umbilical cord blood (UCB) was approved by the Medical Ethical Committee of the Academic Medical Center. Thymocytes were obtained from surgical specimens removed from children up to 3 years of age undergoing open heart surgery and UCB was collected with informed consent of the patients in accordance with the Declaration of Helsinki.

## Author Contributions

MN designed the study, did experiments, analyzed the data, and wrote the manuscript; KG did experiments, analyzed the data, and wrote the manuscript; BB analyzed the data and wrote the manuscript; HS designed the study, analyzed the data, and wrote the manuscript; and the all authors critically read the manuscript.

## Conflict of Interest Statement

The authors declare that the research was conducted in the absence of any commercial or financial relationships that could be construed as a potential conflict of interest.

## Supplementary Material

Figure S1 **(A)** Control or Id2^+^(mCherry^+^) cells cultured on OP9, OP9–Jag1, OP9–Jag2, and OP9–DL1 were gated as CD1a^−^CD3^−^CD4^−^CD8^−^CD94^−^BDCA2^−^ and further analyzed for their expression of CD127, CD161, CD5. c-Kit, CRTH2, and NKp44 expression were analyzed on Id2^+^Lin^−^CD127^+^CD161^+^CD5^+^ cultured on OP9 cells. The data shown here are representative of two independent experiments at day 14. **(B)** Fold cell expansion rate of mCherry^+^Lin^−^CD127^+^CD161^+^CD5^+^ cells. Control (CTRL) or Id2-transduced PNT CD34^+^CD1a^−^ progenitor cells were cocultured with OP9 cells expressing/not expressing Notch ligands [Jagged1 (Jag1) or Jagged2 (Jag2) or Deltalike1 (DL1)] for 2 weeks, the expansion rate was determined at day 7 and day 14 after start of the culture. **(C)** Id2^+^Lin^−^CD127^+^CD161^+^CD5^+^ cells generated on OP9-Jag1 were sorted at day7 and cultured for a further 7 days on OP9 cells with IL-7, SCF, and Flt3L, with or without IL-15, and analyzed for their NK cell markers. Data shown is one representative of two independent experiments. **(D)** CD4 staining of Id2^+^Lin^−^CD127^+^CD161^+^ cells. Data shown are one representative of two independent experiments.

Figure S2 **(A)** Flow cytometry of thymic innate lymphoid cells (ILCs) showing the expression of CD5 and α4β7. **(B)** Flow cytometry of CD161 MACS-enriched cord blood ILCs (red) and T cells (black) showing the expression of CD5. **(C)** qPCR analysis of Id2 and promyelocytic leukemia zinc finger (PLZF) mRNA expression levels in thymic CD34^+^CD1a^+^ cells. NK cells and T cells isolated from the thymus were used as a reference. The data shown are average of three donors.

Figure S3 **(A)** qPCR analysis of IL-2 gene expression level of total PNT CD5^+^ ILC compared to CD5^−^ innate lymphoid cells (ILCs) after P/I stimulation. Tonsil T cells were used as stimulated and unstimulated references. **(B)** qPCR analysis of cytokine mRNA expression levels in adult peripheral blood CD5^+^ ILCs compared to CD5^−^ ILC subsets after P/I stimulation. The data shown are average of four donors. All the qPCR values presented are relative to GAPDH expression.

## Abbreviations

ILC, innate lymphoid cells; Lin, lineage; PNT, postnatal thymus; UCB, umbilical cord blood; SCF, stem cell factor; PLZF, promyelocytic leukemia zinc finger; Id2, inhibitor of DNA-binding protein 2.
