# Supplementary figures and images for: Human CD5+ Innate Lymphoid Cells Are Functionally Immature and Their Development from CD34+ Progenitor Cells Is Regulated by Id2
Source: Front Immunol. 2017 Aug 31;8:1047. doi: 10.3389/fimmu.2017.01047 (PMC5583608; doi:10.3389/fimmu.2017.01047)

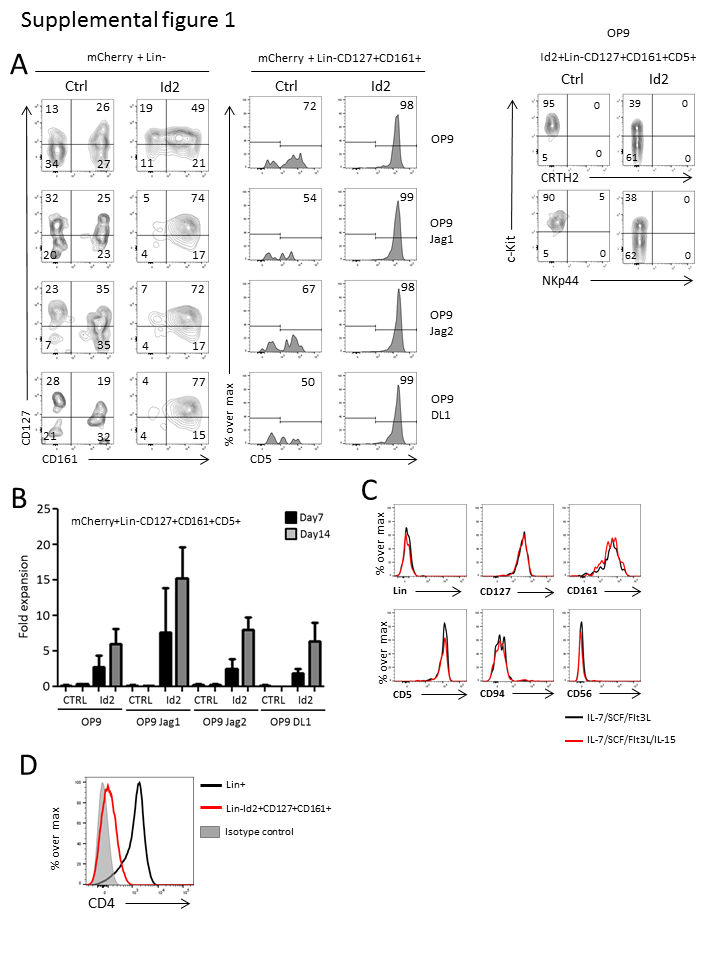

Supplement: Figure S1 — (A) Control or Id2+(mCherry+) cells cultured on OP9, OP9–Jag1, OP9–Jag2, and OP9–DL1 were gated as CD1a−CD3−CD4−CD8−CD94−BDCA2− and further analyzed for their expression of CD127, CD161, CD5. c-Kit, CRTH2, and NKp44 expression were analyzed on Id2+Lin−CD127+CD161+CD5+ cultured on OP9 cells. The data shown here are representative of two independent experiments at day 14. (B) Fold cell expansion rate of mCherry+Lin−CD127+CD161+CD5+ cells. Control (CTRL) or Id2-transduced PNT CD34+CD1a− progenitor cells were cocultured with OP9 cells expressing/not expressing Notch ligands [Jagged1 (Jag1) or Jagged2 (Jag2) or Deltalike1 (DL1)] for 2 weeks, the expansion rate was determined at day 7 and day 14 after start of the culture. (C) Id2+Lin−CD127+CD161+CD5+ cells generated on OP9-Jag1 were sorted at day7 and cultured for a further 7 days on OP9 cells with IL-7, SCF, and Flt3L, with or without IL-15, and analyzed for their NK cell markers. Data shown is one representative of two independent experiments. (D) CD4 staining of Id2+Lin−CD127+CD161+ cells. Data shown are one representative of two independent experiments. [file image_1.tif]

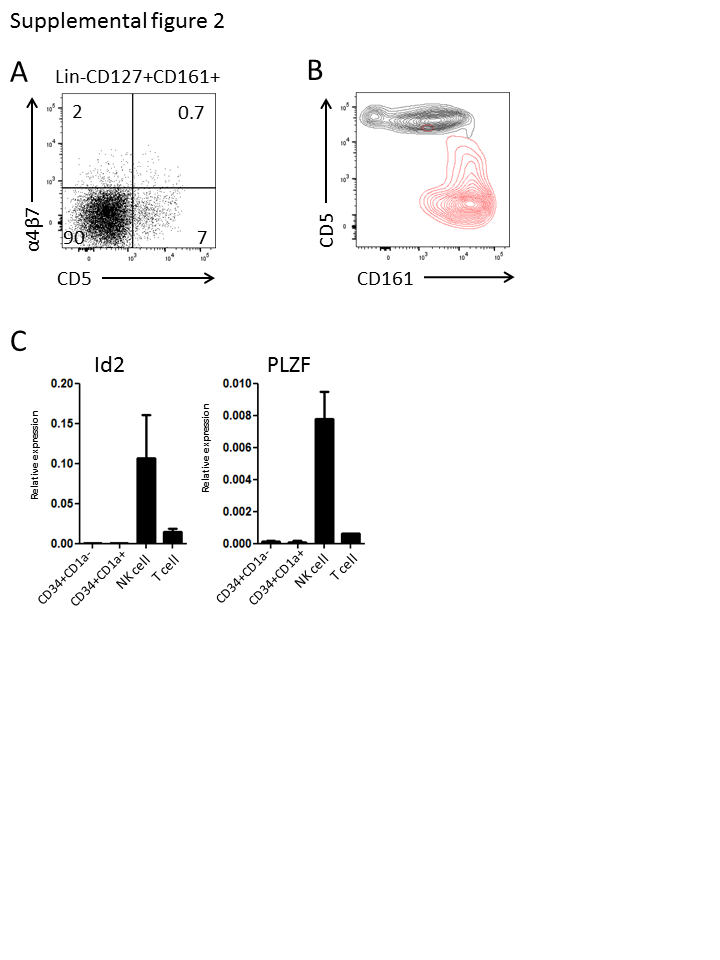

Supplement: Figure S2 — (A) Flow cytometry of thymic innate lymphoid cells (ILCs) showing the expression of CD5 and α4β7. (B) Flow cytometry of CD161 MACS-enriched cord blood ILCs (red) and T cells (black) showing the expression of CD5. (C) qPCR analysis of Id2 and promyelocytic leukemia zinc finger (PLZF) mRNA expression levels in thymic CD34+CD1a+ cells. NK cells and T cells isolated from the thymus were used as a reference. The data shown are average of three donors. [file image_2.tif]

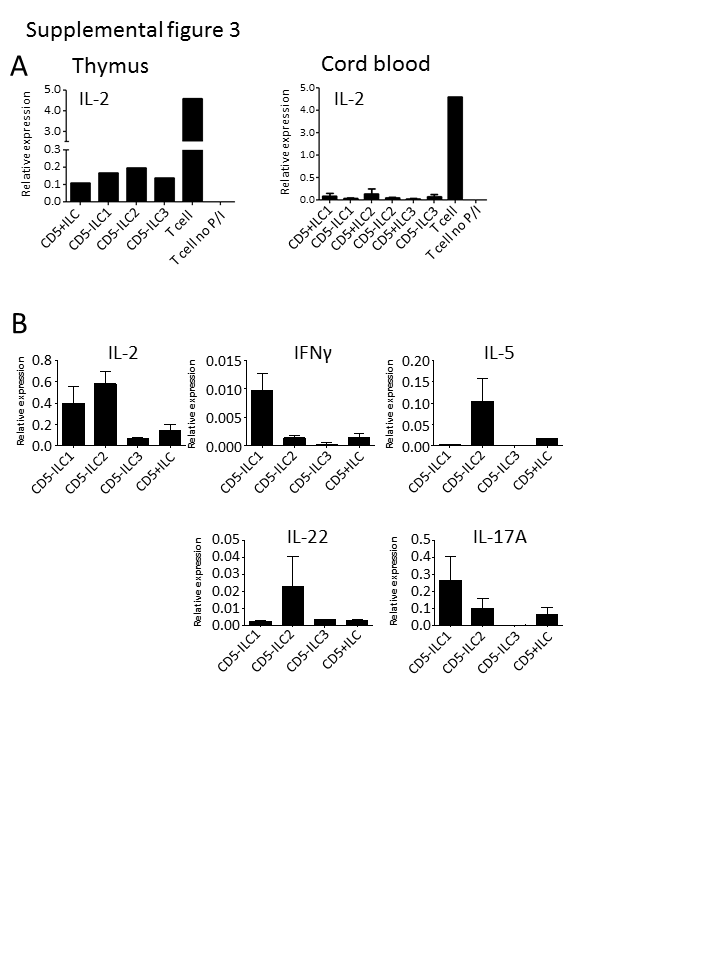

Supplement: Figure S3 — (A) qPCR analysis of IL-2 gene expression level of total PNT CD5+ ILC compared to CD5− innate lymphoid cells (ILCs) after P/I stimulation. Tonsil T cells were used as stimulated and unstimulated references. (B) qPCR analysis of cytokine mRNA expression levels in adult peripheral blood CD5+ ILCs compared to CD5− ILC subsets after P/I stimulation. The data shown are average of four donors. All the qPCR values presented are relative to GAPDH expression. [file image_3.tif]
